# Development and external validation of the NEO-READY model to predict date of discharge among premature neonatal intensive care patients

**DOI:** 10.1038/s41372-026-02827-2

**Published:** 2026-07-20

**Authors:** Hannah Lonsdale, Kevin Patel, Henry Domenico, Ryan S. Moore, Allison B. McCoy, Benjamin French, S. Trent Rosenbloom, Daniel W. Byrne, Robert E. Freundlich, Mhd Wael Alrifai

**Affiliations:** 1 Department of Anesthesiology, Vanderbilt University Medical Center, Nashville, TN, USA.; 2 Department of Neonatal and Newborn Medicine, Kaiser Permanente Roseville Medical Center, Roseville, CA, USA.; 3 Department of Biostatistics, Vanderbilt University, Nashville, TN, USA.; 4 Department of Biomedical Informatics, Vanderbilt University Medical Center, Nashville, TN, USA.; 5 Whiting School of Engineering and the Department of Biostatistics, Johns Hopkins University Bloomberg School of Public Health, Baltimore, MD, USA.; 6 Departments of Anesthesiology and Biomedical Informatics, Vanderbilt University Medical Center, Nashville, TN, USA.; 7 Departments of Pediatrics and Biomedical Informatics, Vanderbilt University Medical Center, Nashville, TN, USA.

## Abstract

**OBJECTIVE:**

To develop a parsimonious, interpretable, and accurate model for predicting discharge for premature infants in the NICU—a model suitable for prospective evaluation and integration into clinical workflows.

**STUDY DESIGN:**

Using routinely available electronic health record data, we developed and validated NEOnatal Reliable Estimation of Approaching Discharge in Young Infants (NEO-READY), a daily-updating model that predicts the likelihood of discharge within 5 days for premature infants.

**RESULTS:**

Data from 702 infants were used to develop the model, and data from 201 infants were used for temporal external validation. The model included 13 predictors and two interaction terms and demonstrated excellent discrimination across development (AUC = 0.88, 95% CI 0.87–0.90) and validation (0.90, 0.88–0.91) cohorts.

**CONCLUSION:**

This work represents step 1 toward our long-term goal: integrating the NEO-READY model into clinical workflows as part of a comprehensive strategy to improve discharge preparedness, reduce discharge delays, and optimize NICU resources.

## INTRODUCTION

Premature infants admitted to the neonatal intensive care unit (NICU) often experience prolonged and medically complex hospitalizations due to associated complications such as intraventricular hemorrhage, retinopathy of prematurity, bronchopulmonary dysplasia, and sepsis [1]. These infants account for over half of NICU admissions, and more than 40% require surgical intervention during their stay, further extending hospitalization [1]. Discharge from the NICU is a highly complex, resource-intensive process requiring structured and coordinated planning from a multidisciplinary team that includes physicians, nurses, social workers, and case managers. Proactive timeline management with accurate forecasting of the likely date of readiness for discharge allows the multidisciplinary team to ensure that tasks such as caregiver training, follow-up arrangements, and screening examinations are completed to coincide with the infant’s physiological readiness for discharge [2]. Failure to accurately communicate, collaborate, and anticipate discharge readiness can lead to the patient reaching physiological readiness for discharge before discharge planning is completed. This may prolong hospitalization unnecessarily, consuming scarce NICU capacity and increasing healthcare costs, rates of preventable hospital readmissions, and care-related failures [3].

Despite the operational, financial, and clinical importance of timely discharge, the burden and causes of discharge delays in premature infants are not extensively reported. To our knowledge, there has been only one study that examined the incidence of delayed discharge from the NICU to home; this study found 116 documented delays accounting for 480 lost bed-days over 2 years in a single unit. Reported reasons for delay that could be preventable with better discharge anticipation included provision of home services and equipment, clearance for discharge from visiting services, difficulties contacting the family member responsible for discharge, and coordination of hearing and visual tests. Communication failures between medical staff and discharge coordinators were cited as a major challenge[4]. Families share this burden: when discharge preparation is backloaded, rushed, or disorganized, parents often feel overwhelmed and underprepared, which correlates with less confidence in their caregiving abilities and higher rates of home care errors involving feeding, medications, and oxygen equipment. Conversely, initiating continuous, systematic education earlier in the NICU stay—as the patient begins to approach physiological discharge readiness—improves parental mastery of technical skills, emotional comfort, and overall discharge readiness [2, 5, 6]. Accurate prediction of physiological readiness for discharge could improve communication across multidisciplinary teams and with caregivers, leading to earlier initiation of complex discharge planning tasks and caregiver education, thereby improving preparedness and reducing anxiety. Preventing delayed discharges would additionally increase NICU resource availability for admission of new patients.

Most published models focus on estimating NICU length of stay or identifying infants at risk of prolonged hospitalization, but have limited potential applications to clinical practice and/or discharge preparation. Existing models are often designed only to predict length of stay on the day of admission [7–16], have exclusions that render them unsuitable for prospective use [11, 12, 16–18], or use data that would not be available during the patient’s admission, such as billing codes [19, 20]. Temple and colleagues [17] used progress note data and machine learning to predict discharge within 2–10 days. Their models achieved the highest performance at 2 days before discharge, with an area under the curve (AUC) of 0.854. However, a 2-day prediction window does not allow sufficient time to complete discharge planning for complex patients. Finally, only two published studies report external validation and prospective testing [14, 21], and none report measurable impact on patient care or progression to clinical implementation.

The objective of this study was to develop and validate a prognostic model that can be applied daily to generate a dynamic score on each day of admission, indicating the probability of discharge within the next 5 days for premature infants in the NICU. By using readily available data to produce updated daily predictions and by accurately predicting discharge within a 5-day window, the model addresses limitations of prior approaches and aims to facilitate the timely initiation of discharge planning. Our long-term goal is to integrate this model into the NICU electronic health record workflow as part of a collaborative strategy to improve infant, parent, and care team preparedness; reduce discharge delays; and optimize NICU care capacity.

## METHODS

### Study population

This study used retrospective electronic health record (EHR) data from patients born between 28 and 37 weeks of gestation who were admitted for active care to a Vanderbilt University Medical Center (VUMC) NICU after November 2, 2017, and discharged before January 01, 2020. No further inclusion or exclusion criteria were applied. The study period was chosen to begin with the implementation of our current EHR [Epic, Epic Systems Corporation, Verona, Wisconsin, USA] and to end before the start of the COVID-19 pandemic, due to concerns of an exceptional impact on discharge practices and coordination. The development cohort consisted of 702 premature patients who met inclusion criteria and were admitted for active care to the VUMC NICUs between November 2, 2017, and July 1, 2019. The temporal external validation cohort consisted of 201 premature patients admitted for active care to the VUMC NICUs between July 2, 2019, and discharged before January 01, 2020. All hospital identifiers were removed, and patients were assigned a unique study identifier to protect privacy. This study was approved with a waiver of informed consent by the Vanderbilt University Medical Center Institutional Review Board (IRB) [LONSDAH09192024120046]. The study involved a retrospective secondary use of data collected for routine clinical care, and therefore the IRB considered the study to involve no more than minimal risk to participants.

The VUMC NICUs comprise 119 beds across three geographic areas within the medical center: two private-room units in a free-standing children’s hospital and a third open-bay unit adjacent to the labor and delivery area within the adult hospital. The VUMC NICUs admit approximately 1500 infants annually and serve as a regional perinatal referral center with approximately 40% of patients transferred from other facilities (outborn). The patient population includes preterm and term infants, including those with complex surgical and cardiac anomalies. In keeping with standard practice at most US institutions, infants are routinely discharged directly home from the NICU without transition to stepdown units [22].

### Data collection and processing

We obtained retrospective data from the EHR reporting database (Clarity). Data were processed using Structured Query Language (SQL) to create fields containing one observation per day for each patient. The dataset was then screened for missing and physiologically implausible values. The latter were removed from the dataset before further processing and treated as missing. Physiologically implausible values may be introduced as typographical errors when clinical staff manually enter data in a patient’s chart. Wherever possible, missing values were then filled by manual chart review. The degree of true missingness (after chart review) for each variable is reported in Table 1. For vital signs and feeding intake, within-patient last-observation-carried-forward was used to impute missing daily values. Median value imputation was used in cases of first-day missing data for continuous variables or an “Unknown” category in cases of categorical variables.

### Outcome

The prognostic model was developed using a discrete-time framework with data at the patient-day granularity. The binary outcome was defined as discharge within 5 days of the prediction date. A 5-day window was considered sufficient time to complete discharge planning for a majority of patients, while allowing enough to be known about the patient’s clinical condition to result in a reliable prediction. Patients were censored in the event of death or discharge to a location other than home. To prevent information leakage, once a patient met outcome criteria (discharge within 5 days), subsequent inpatient days were excluded from the analysis.

### Potential predictors

A group of neonatology experts identified relevant features associated with readiness for discharge from published literature and their clinical experience. The list of relevant features was reviewed, pruned, and supplemented in collaboration with informaticians to select only fields reliably available on each patient day. Features were included as candidate predictors if they were available during the hospital admission and could be used to update the prediction daily. Forty candidate predictors (Supplementary Table 1) were assessed. Variables were selected based on their daily availability in the chart, reliability, prior evidence supporting their association with length of stay, clinical plausibility, and their potential to improve model performance. With 638 events in the development cohort, we calculate 16.0 events per candidate degree of freedom, which is sufficient to prevent overfitting[23].

### Statistical analysis

Variables were summarized as counts and percents for categorical variables and median with interquartile range (IQR) for continuous variables. Univariate associations were tested for using the Mann-Whitney U test for continuous variables and Pearson’s chi-square test for categorical variables. Because data are structured as one observation per patient-day, rather than a continuous time interval, a discrete-time survival model with a binary logistic regression classifier [24] was used. Updated information from every 24-h period during a patient’s admission was used by the model to generate a dynamic score indicating the probability of discharge within the next five days for premature infants in the NICU. Two interaction terms (birth location and day of hospitalization, birth location and gestational age at birth) and one non-linear term (day of hospitalization) were clinically prespecified for our modeling approach. Further nonlinear terms were not evaluated due to sample size limitations and the absence of prior evidence supporting their necessity. LASSO (least absolute shrinkage and selection operator) with 10-fold cross-validation [25] was used for final variable selection. After variable selection, the final model was fit using a standard generalized linear model. Table 1 lists the variables that were used in the final model.

The prognostic accuracy of the final model was evaluated using measures of discrimination (Area Under the Receiver Operating Characteristic Curve (AUC) and Area Under the Precision-Recall Curve (AUPRC)) and calibration (calibration intercept and slope, visual examination of the calibration curve, and Brier score). In accordance with TRIPOD guidelines [26], we performed an external temporal validation using a non-overlapping cohort admitted in a later time period and evaluated discrimination and calibration without model updating. We acknowledge that geographic validation is a necessary next step and is planned. Model performance was evaluated in both the development and the temporal external validation cohorts. Variable importance was reported using each predictor’s chi-square test statistic from the final fitted model. Within the validation cohort, Youden’s cut point was used to determine the optimal risk cutoff for binary decision rules at 5-day intervals during each patient’s admission. Model sensitivity, specificity, positive predictive value (PPV), and negative predictive value (NPV) were reported using the highest Youden index as a threshold for each time point.

A 2-sided p-value of <0.05 was used to indicate statistical significance. No adjustments were made for multiple comparisons. All analyses were conducted using R version 4.4.0. Reporting followed the Transparent Reporting of a multivariable prediction model for individual prognosis or diagnosis (TRIPOD) reporting guidelines for prognostic studies [26]. Elements of the PROBAST tool [27] to assess risk of bias and the applicability of prediction model studies were incorporated to facilitate future assessment of fairness and applicability.

## RESULTS

### Patient characteristics

The 702 infants in the development cohort (admitted between November 2, 2017, and July 1, 2019) contributed a total of 17,842 patient-days. The 201 infants in the validation cohort (admitted between July 2, 2019, and discharged before January 1, 2020) contributed 4587 patient-days and were used to temporally validate the model. The characteristics of the patients in each cohort are summarized in Table 2. A total of 85 (9.4%) patients either died or were back-transferred to other NICUs and were therefore treated as censored. Patients with lengths of stay <5 days (49/903, 5.2%) were included in the cohort. The longest admission was 243 days, and the shortest was 1 day. Race and ethnicity, while not used as inputs to the prognostic model, were missing or unknown for 7.2% and 60.4% of patients, respectively. All other patient characteristic fields did not have missing data.

### Model description and performance

The final logistic regression model included 13 variables and two interaction terms (Fig. 1; Supplementary Table 2, https://cqs.app.vumc.org/shiny/NICUDischargePrediction). The variables most strongly associated with discharge within 5 days were, in order of importance, percentage of nutritional intake taken orally for the past 24 h, highest environmental temperature for past 24 h, day of hospitalization on which the prediction was made, gestational age at birth, and birth location (inborn/outborn). The final model demonstrated excellent discrimination (AUC, 0.88; 95% CI, 0.87–0.90). Calibration was strong with some miscalibration at higher quantiles of predicted risk (Brier score = .036, Fig. 2A). Day-by-day model performance (Table 3) was exceptional.

### Temporal external validation

No significant differences were found in demographic characteristics between the validation and derivation cohorts (Table 2). The model generated from the development cohort was applied to the temporal validation cohort without recalibration, showing excellent discrimination (AUC, 0.90; 95% CI, 0.88–0.91) and calibration, again with some expected miscalibration at higher quantiles of predicted risk (Brier score = 0.039, Fig. 2B).

## DISCUSSION

We achieved our study’s objective by using temporally separate retrospective datasets to develop and externally validate an accurate prognostic model that can be applied daily to generate a dynamic score indicating the likelihood of discharge within the next 5 days for premature infants in the NICU. The final model, a discrete-time survival model with a binary logistic regression classifier, demonstrated excellent overall discrimination and satisfactory calibration. Input variables were included only if they were readily available and regularly updated in the EHR, to support dynamic, near-real-time predictions. The use of a parsimonious set of 13 input variables makes the model feasible for future automated use at the point of care. Minimal exclusion criteria and censoring patients who were not discharged home, rather than excluding them, also readies the model for future clinical implementation.

The model’s overall performance in the validation cohort is very strong (AUC = 0.90, 95% CI 0.88–0.91), and discrimination improved still further when measured on a day-specific basis (Table 3). This may be because, at the same time during their hospital admission, infants are at a more homogeneous stage of illness, while the overall AUC averages performance over all time points and therefore represents a more heterogeneous group of clinical conditions. At this stage of model development, selection of a single hard cut point leading to simple dichotomization such as “likely to discharge” vs. “not likely to discharge” would lead to significant information loss [28]. Although in time this may be valuable and could be used, for example, to trigger prompts for discharge planning tasks, any such cut point would require extensive clinical testing and is therefore beyond the scope of the current study.

The model generally shows acceptable calibration across the range of clinically relevant risk levels (Fig. 2). In the validation cohort, higher predicted probabilities tended to overestimate the observed event rate, which is not unusual given the limited number of high-risk observations. Clinically, management in this group does not depend on small differences within this range; therefore, modest over-prediction is unlikely to influence decision-making. We have implemented the model as a Shiny application at https://cqs.app.vumc.org/shiny/NICUDischargePrediction.

Poor alignment in anticipation of discharge readiness across the multidisciplinary team and failure to complete non-medical preparation before physiological readiness can lead to unnecessarily prolonged hospitalization, which is associated with increased caregiver anxiety [29], excess morbidity and mortality [30], hospital-acquired infections [31], adverse medication events [31], increased cost, and blocking of finite hospital care resources. We intend for our model to support team communication and a shared mental model for anticipation of physiologic readiness for discharge. More coordinated anticipation of discharge could improve family preparedness, trigger earlier initiation of complex discharge tasks, and enhance NICU resource availability.

Our model is ideally suited for prospective clinical use due to the following characteristics:

- Feasibility—Uses common and readily available EHR data that can be automatically extracted without additional user input. The use of a parsimonious model based on logistic regression, rather than a more complex technique such as machine learning, reduces the number of input values needed to produce a daily prediction and the computing power needed to execute real-time predictions, making daily use at the point of care very feasible, in the experience of members of this author team [32–37]. Input variables were selected for inclusion only if available at the point of daily clinical assessment. During daily assessments, we also cannot know which patients will survive or will require back-transfer; therefore, those patients were included but censored in the model, rather than excluded from the dataset.
- Clinically reasonable—Developed in full collaboration between modeling experts and clinical neonatologists. As a result, all variables have strong clinical validity. A review of the model predictors and their coefficients shows that the first and second most significant predictors, percentage of intake taken orally and highest environmental temperature in the past 24 h, have the largest contribution to the likelihood of discharge. The ability to take full feeds and to maintain body temperature within external warming devices represents two important criteria for discharge.
- Applicability—The inclusion criteria encompass a broad demographic of NICU patients known to be at risk of requiring longer stays and more complex discharge planning, and further widening of the target population is planned for future work. Patients with lengths of stay less than 5 days were intentionally included, despite the potential for negative impact on model performance, as they comprise an essential population of any level III or IV NICU.
- Dynamic prediction—Designed to provide continual assessment of each patient’s discharge readiness, updated early in the morning each day with the patient’s most current clinical information to generate a likelihood of discharge within 5 days. It is not limited to a specified time or set of input features.
- Actionable prediction window—The model produces excellent discrimination for a clinically valuable prediction window. A 5-day prediction window was chosen as an ideal balance between statistical performance and clinical utility, giving sufficient lead-time to complete discharge planning tasks for most patients.
- Rigorously validated—A dataset from patients with temporally separate admission dates from the development cohort was used for external validation. The model demonstrated excellent performance on this external validation.

An extensive literature search found several reports of models developed to predict NICU length of stay (Supplementary Table 3). However, only one of these models [17] was specifically designed for discharge planning. Temple and colleagues [17] used supervised machine learning to predict days to discharge (DTD) for patients in the NICU; however, they did not report validation beyond the initial training dataset, and they used ICD-9 diagnostic codes as model inputs. ICD-9 codes are assigned only after discharge, so they would not be available to generate real-time predictions. In keeping with information that is likely to be of high clinical importance, our model had several predictors in common with that of Temple and colleagues, including percentage of oral intake, weight, gestational age at birth, administration of caffeine, and number of cardiopulmonary events within the past 24 h. However, our model used additional elements, including environmental temperature, birth location (inborn/outborn), laboratory measurements, and average mean arterial pressure (MAP) within the past 24 h. We included the total number of medications administered, whereas Temple and colleagues limited their count to intravenously administered medications only. Our model demonstrates improved performance over the Temple model’s predictions for 4 DTD and 6 DTD (AUCs 0.795 and 0.754, respectively). There are several possible reasons for this. (1) Environmental temperature was not used by Temple and colleagues but was the second strongest predictor in our model. (2) Temple and colleagues excluded patient-days with missing data rather than filling missing data fields. As patients recover and become closer to discharge, they are less closely monitored and so are perhaps more likely to have missing data in their chart. Exclusion of these patient-days may have reduced the training data available to their model, with subsequent impact on model performance. (3) We included variables that could be considered proxy measures of patient complexity (outborn birth location, total number of medications) or ongoing acute pathology (laboratory measurements, MAP).

Most other published models [7–16] were designed to predict length of stay only at NICU admission. These retrospective, static models are restricted to input data available at admission and therefore cannot account for evolving clinical events that can significantly impact a patient’s length of stay. Such events include sepsis, necrotizing enterocolitis, or failure to wean from respiratory support. Unsurprisingly, all models trained solely using admission data are limited in the granularity of their predictions and therefore have little clinical utility for predicting discharge readiness in individual patients. Additionally, several models are unsuitable for prospective clinical use because they rely on factors such as billing codes, which would not be available at the point of care [19, 20]. Others exclude a large proportion of patients, including those who underwent surgery, were outborn, backtransferred, or had congenital abnormalities [11, 12, 16, 18, 38]. Several studies excluded patients with missing data, rather than attempting chart review or imputation [7, 17, 39], and one used manually collected data [21]. These failings make prospective real-time use impractical or impossible. Finally, our proposed model is one of the few studies found to demonstrate model validation beyond the original dataset [14, 21].

### Limitations

Despite very strong model performance and careful attention to preparation for future clinical use, there are important limitations to consider. The retrospective datasets were collected from a single institution; therefore, the model may not be generalizable to other centers or to present discharge practices at our own institution. Although discharge guidelines are published, each hospital has its own practice and discharge threshold for preterm neonates, which evolves over time and may affect the model’s performance. As with all prognostic models, prospective validation and recalibration as needed for new domains is vital. The model was also trained using actual discharge dates, rather than the date of physiological readiness for discharge. Physiological readiness status is not explicitly recorded at many institutions, including ours. Some patients may have been ready for discharge earlier than their actual discharge date. Therefore, the model may perform even better if we can adjust for patients who experience nonmedical delays in discharge. This would be achieved by retraining with the date of physiological readiness for discharge as the predictive end point, rather than use of date of actual discharge as in the current model. However, many institutions do not routinely record discrete “readiness for discharge” status for each patient and so that information would not be available to test the model’s performance (geographic validation) prior to deployment at that institution. There may also be value in using both models together to detect patients at high risk of delayed discharge, who may have a significant difference between the two outputs.We trained this initial model to predict discharge for babies born at 28 to 37 weeks of gestation. This population was chosen as an early focus for the project due to their mid-level complexity, with a somewhat more predictable LOS than extremely premature infants, but still with significant variability. This population is likely to need significant post-discharge care planning but not so complex that it cannot be completed in most cases within the five-day window of prediction. Extremely premature infants (born at less than 28 completed weeks of gestation) frequently have very complex discharge needs and a need for durable medical equipment at discharge; therefore, the 5-day predictive window of the current model may not be sufficient to complete discharge preparation. Term babies were not included as many are admitted for fewer than 5 days; therefore, the model is of limited clinical utility for most patients in this population. There is, however, a subset of term infants with complex needs who experience longer stays and may benefit from better discharge forecasting. Our current model represents a promising start to a wider project to improve discharge planning in the NICU. Future work will address extending the prediction window to assist multidisciplinary anticipation of discharge for patients with more complex needs (e.g., durable medical equipment), geographical validation, and exploratory domain validation for extremely preterm and complex term babies.

The model’s discrimination is excellent for lengths of stay of 10 to 40 days (Table 3). Before 10 days, clinical presentations are at maximum heterogeneity. Model performance may be improved by removing patients with a length of stay of less than 5 days, but with prospective use, we could not foresee with certainty which patients would be discharged within 5 days. Complex discharge planning needs are more likely in infants with a length of stay >10 days; therefore, predictions are more useful in this group than for those with shorter admissions. After 40 days, only around 10% of patients remain admitted, limiting the size of the dataset that can be used to train the model. The addition of data from more patients with long stays, or the creation of synthetic training data, may improve model performance on this ‘long tail’. Model performance may be further improved by inclusion of slope of change parameters, and this will be investigated in future work.

Selection of model inputs was limited by the availability of structured data within the EHR. Some data points were missing for daily weight, oral intake, and MAP. Although we used established imputation methods, missing data may still introduce bias or inaccuracies in the model. All charted information was accurately transferred to the modeling process, but some EHR data capture is performed by direct observation and so carries the potential for inaccuracy. It can be especially challenging to accurately capture the volume of oral intake during breastfeeding. We therefore used a percentage of total intake, rather than an absolute volume. For clinical implementation, we plan to include imputation methods that can provide the most appropriate real-time replacement for missing values. Completion of race and ethnicity fields is optional in our EHR, and they were not completed for 7.2% and 60.4% of patients, respectively. These fields were not used as input variables, but appraisal of these demographics should be undertaken cautiously for the cohorts.

## CONCLUSION

Despite these potential limitations, the product of our development and validation work is a very strong model to predict discharge from the NICU within 5 days, with clear potential for successful prospective assessment and future implementation. Our model overcomes many limitations of previous work to predict the length of stay or discharge readiness for NICU patients. Work is underway to record the date of readiness for discharge for patients at the VUMC NICUs, and this will allow recalibration to predict the date of a patient’s physiological readiness for discharge rather than the actual discharge date. Future work will address prospective temporal validation and exploratory domain validation for complex term and extremely preterm babies, combined with initiatives to embed predictions into the EHR and to limit missing and physiologically implausible data at the point of capture. We will then demonstrate potential clinical value by directly comparing the model’s predictions to predictions of discharge readiness from attending neonatologists.

Our long-term goal is to integrate this model into NICU workflows as part of a comprehensive strategy to improve infant, parent, and care team discharge preparedness; reduce discharge delays; and optimize NICU care capacity. Successful and sustained improvement in timely patient discharge requires not only excellent forecasting but also action to comprehensively identify and overcome barriers in the current healthcare system. We look forward to further collaborations as we undertake future mixed-methods work leading to a pragmatic randomized controlled trial and clinical implementation.

## Supplementary Material

Supplemental content

ADDITIONAL INFORMATION

**Supplementary information** The online version contains supplementary material available at https://doi.org/10.1038/s41372-026-02827-2.

## Figures and Tables

**Fig. 1:**
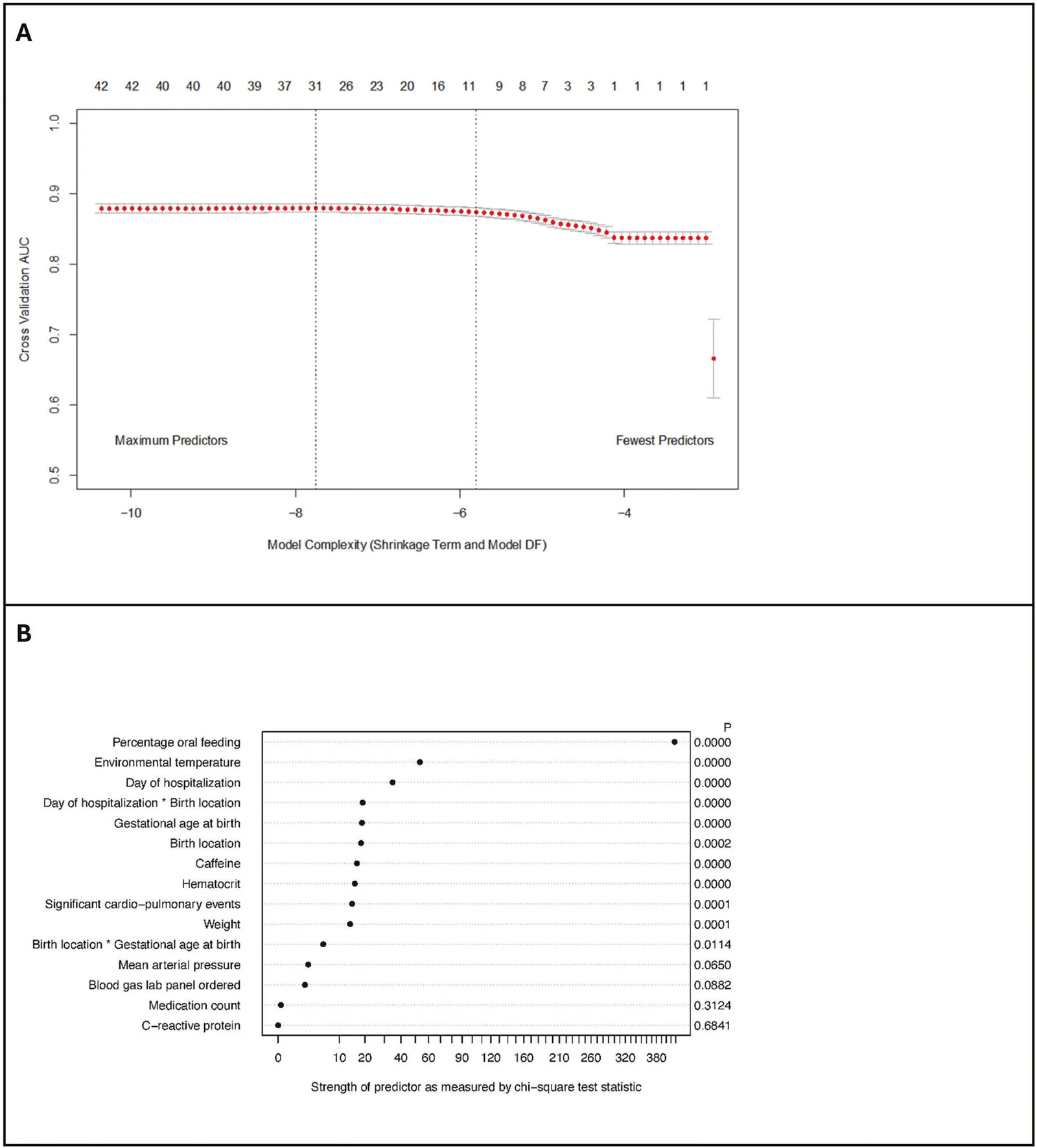
Model performance. **A** Cross-validation model accuracy vs. model complexity. Accuracy is measured as cross-validation AUC with a 95% confidence interval. The bottom x-axis shows the LASSO shrinkage term. The top x-axis shows the model degrees of freedom. **B** Predictors included in the final model were ranked by the strength of the predictor, as measured by the model chi-square test statistic. P-values derived from chunk testing of the term and subsequent interaction. *AUC* area under the receiver operating characteristic curve, *DF* degrees of freedom, *LASSO* least absolute shrinkage and selection operator.

**Fig. 2:**
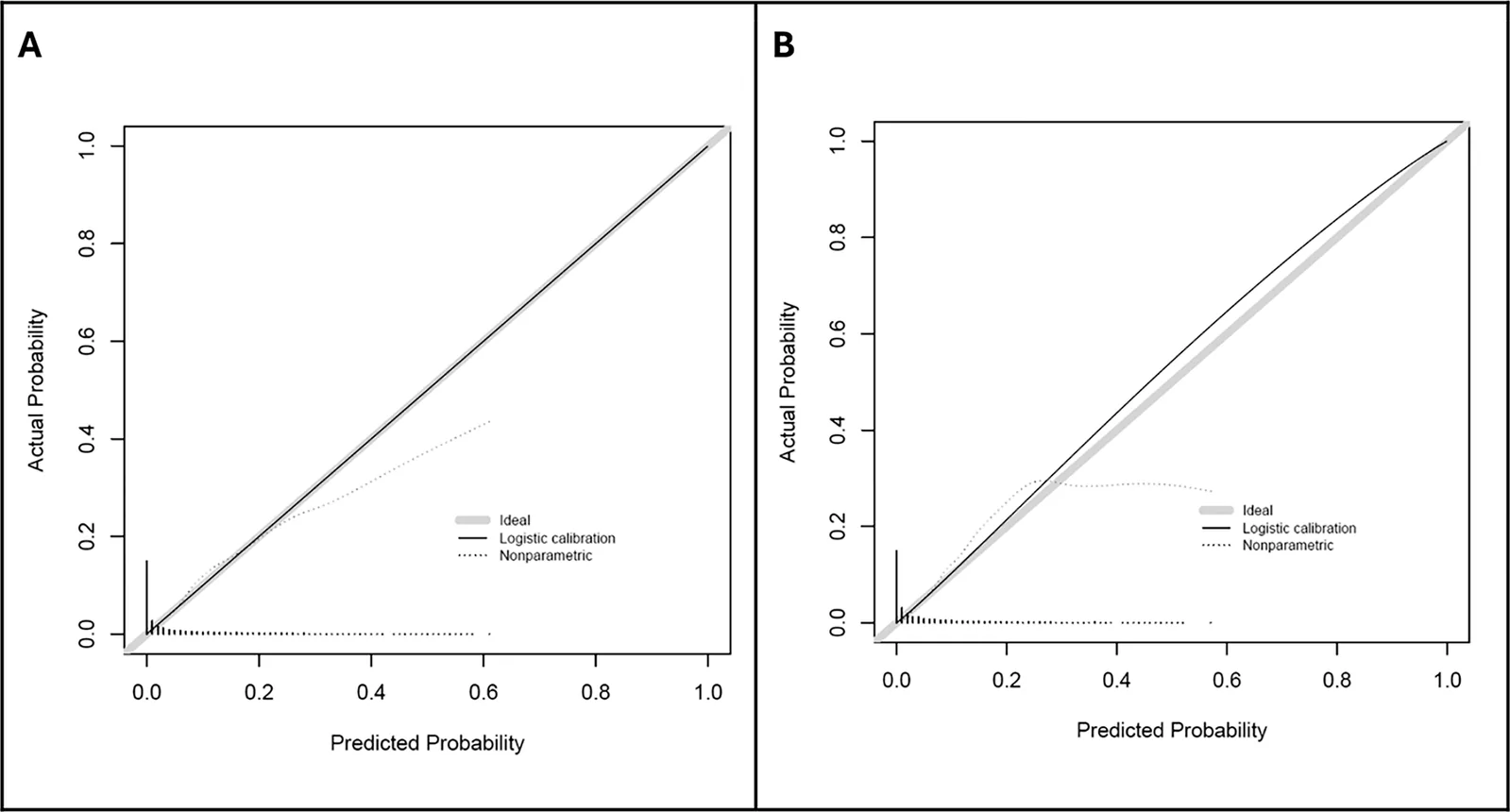
Model Calibration Curves. Calibration curves for **A** build cohort and **B** validation cohort.

**Table 1.** Variables used in the final model.

Input feature	Source	Degree of missingness	Definition
Gestational age at birth (weeks + days)	Administration	0	Time since mother’s last menstrual period
Birth location (inborn/outborn)	Administration	0	Born at VUMC (inborn) or transferred to VUMC after birth (outborn)
Current day of admission	Administration	0	Number of days since admission
Daily weight (g)	Flowsheets	7.4%	Maximum recorded weight in the past 24 h
Average daily mean arterial pressure (MAP) (mmHg)	Flowsheets	1.7%	Daily mean of documented MAPs in the past 24 h
Percentage oral intake (%)	Flowsheets	7.2%	Percentage of documented volume of breastmilk and/or formula administered orally in the past 24 h
Highest environmental temperature (°C)	Flowsheets	0	Maximum incubator set temperature, or room temperature (26°C) if nursed in an open crib in the past 24 h
Caffeine administered (yes/no)	Medication administration record	0	Yes, if oral or intravenous caffeine was administered in the past 24 h
Significant cardio-pulmonary events (count/day)	Flowsheets	0	Count one when any of the following occur in the past 24 h: • Heart rate <80 for >5 seconds • Oxygen saturation <85 for >5 seconds • Apnea for >20 seconds • Recorded intervention, any of: moderate or vigorous tactile stimulation, PPV, CPAP, blow-by oxygen …and *not* during feeding, suctioning, or gastric tube placement
CRP measured (yes/no)	Laboratory results	0	Yes, if any C-reactive protein result in the past 7 days
Blood gas measured (yes/no)	Laboratory results	0	Yes, if any blood gas result in the past 7 days
Medications administered (count)	Medication administration record	0	Number of unique medications administered in the past 24 h
Hematocrit measured (yes/no)	Laboratory results	0	Yes, if any hematocrit result in the past 7 days

Features are updated daily at midnight with the previous calendar day’s information. Missingness reported at a patient-day level. *VUMC* Vanderbilt University Medical Center, *MAP* mean arterial pressure, *PPV* positive pressure ventilation, *CPAP* continuous positive airway pressure, *CRP* C-reactive protein.

**Table 2.** Patient characteristics and demographics.

Characteristic	Sub-categories	Overall (*N* = 903)	Development cohort (*N* = 702)	Validation cohort (*N* = 201)
Length of stay (days)		19 (12–32)	19 (11–33)	19 (13–27)
Gestational age at birth (weeks)		33 (32–35)	33 (32–35)	34 (32–34)
Birthweight (g)		1921 (1550–2340)	1920 (1540– 2380)	1950 (1660– 2250)
Sex	Male	462 (51.2%)	362 (51.6%)	100 (49.8%)
	Female	441 (48.8%)	340 (48.4%)	101 (50.2%)
Transferred in from another hospital (outborn)		233 (25.8%)	187 (26.6%)	46 (22.9%)
Race	White	616 (68.2%)	489 (69.7%)	127 (63.2%)
	Black	182 (20.2%)	135 (19.2%)	47 (23.4%)
	Asian	6 (0.7%)	6 (0.9%)	0 (0%)
	Other	34 (3.7%)	20 (2.8%)	14 (7.0%)
	Unknown	65 (7.2%)	52 (7.4%)	13 (6.5%)
Ethnicity	Hispanic	112 (12.4%)	84 (12.0%)	28 (13.9%)
	Not Hispanic	246 (27.2%)	190 (27.1%)	56 (27.9%)
	Unknown	545 (60.4%)	428 (61%)	117 (58.2%)
Preferred language	English	812 (89.9%)	635 (90.5%)	177 (88.1%)
	Spanish	64 (7.1%)	47 (6.7%)	17 (8.5%)
	Other	27 (3.0%)	20 (2.8%)	7 (3.5%)
Died or transferred to a location other than home (censored)		85 (9.4%)	64 (9.1%)	21 (10.4%)

Categorical data are shown as the number of patients (percentage). Continuous data are shown as the median value (interquartile range).

**Table 3.** Model performance based on the validation set.

Day of admission	Patients observed (*N* = 201)	AUC	AUPRC	Highest Youden index	PPV/NPV	Sensitivity/specificity
5	195	0.94	0.61	0.82	0.39/1.0	1.0/0.82
10	171	0.97	0.81	0.87	0.65/0.99	0.96/0.90
15	139	0.96	0.92	0.81	0.75/0.98	0.95/0.85
20	92	0.92	0.77	0.79	0.79/0.95	0.88/0.91
25	64	0.96	0.89	0.83	0.86/0.95	0.9/0.93
30	45	0.94	0.87	0.76	0.60/1.0	1.0/0.76
35	33	0.92	0.76	0.86	0.79/1.0	1.0/0.86
40	22	0.93	0.62	0.88	0.71/1.0	1.0/0.88

*AUC* area under receiver operating characteristic curve, *AUPRC* area under precision-recall curve, *PPV* positive predictive value, *NPV* negative predictive value.

## Data Availability

The data underlying this study were derived from electronic health records and contain protected health information. As such, they are not publicly available. Access to de-identified data may be granted upon reasonable request, subject to institutional review and data use agreements. An interactive version of the model can be viewed as a Shiny app at https://cqs.app.vumc.org/shiny/NICUDischargePrediction.
