# Supplementary material for: Development and external validation of the NEO-READY model to predict date of discharge among premature neonatal intensive care patients
Source: J Perinatol. Author manuscript; Available in PMC 2026 Nov 16. (PMC13773358; doi:10.1038/s41372-026-02827-2)
Supplement: Supplementary file 1 [file NIHMS2219206-supplement-Supplemental_content.pdf]

Supplementary Information

Supplementary Table 1

Overview of extracted features

Supplementary Table 2

Full model and coefficients

Supplementary Table 3

Comparison of current neonatal intensive care unit length of stay and discharge prediction models according to elements of implementable models

Supplementary Table 1. Overview of extracted features.

**Source**	**Feature**
Demographics	Gestational age at birth, post-menstrual age, sex, ethnicity, race, preferred language
Administration	Admission date/time, discharge date/time, length of stay, discharge status
Flowsheets	Birth weight, daily weight, length, head circumference, near-infrared spectroscopy (NIRS) readings, oxygen saturation, body temperature, heart rate, mean arterial pressure (MAP), respiratory rate, form of respiratory support, total intake, total output, volume of formula taken orally, volume of breast milk taken orally, formula by feeding tube, breast milk by feeding tube, incubator set temperature, presence of cooling device, set temperature of cooling device, need for tactile stimulation, NPO status
Medication administration record	Caffeine, number of medications administered
Laboratory and imaging results	White cell count, red cell count, hemoglobin, hematocrit, platelets, blood culture, sodium, potassium, bicarbonate, blood urea nitrate, creatinine, calcium, glucose, troponin, echocardiogram
Clinical notes	Social work, cardiology, neurosurgery, operative report

NPO nil per os.

Supplementary Table 2. Full model and coefficients.

**Model Variable**	**Coefficient**	**P-value**
Percent of nutritional intake taken orally	3.2327	<0.0001
Environmental temperature	−0.1498	<0.0001
Gestational age at birth	0.1675	<0.0001
Birth location	5.3529	0.004
Caffeine administered	−1.2807	<0.0001
Hematocrit measured	−0.4354	<0.0001
Significant CPE	−0.0342	<0.0001
Daily weight	0.0004	0.0001
Birth location: outborn and Gestational age at birth interaction	-0.1332	0.0114
Mean daily MAP	0.0112	0.065
Blood gas measured	−0.2702	0.088
Medication count	−0.0261	0.312
CRP measured	0.0572	0.684

The model additionally includes terms for non-linear day of hospitalization and interaction between non-linear day of hospitalization and birth location.

Coefficients for non-linear terms are not directly interpretable, so these terms are not included in the table.

CPE cardiopulmonary events, *MAP* mean arterial pressure; *CRP* C-reactive protein

Supplementary Table 3. Comparison of current neonatal intensive care unit length of stay and discharge prediction models according to elements of implementable models.* (Arranged in reverse chronological order and limited to publications since 2010.)

**Lead author**	**Year**	**Feasibility**	**Broadly applicable**	**Dynamic risk score**	**Actionable criteria**	**Rigorously validated**
Arkin [1]	2024	🟢	🔴	🔴	🔴	🔴
Erdogan Yildirim [2]	2024	🔴	🟡	🔴	🔴	🔴
Frostig [3]	2023	🟢	🟡	🔴	🔴	🟡
Kermani [4]	2023	🔴	🟡	🔴	🔴	🟢
Wang [5]	2022	🔴	🟡	🔴	🟢	🟡
Singh [6]	2021	🔴	🟡	🟢	🟡	🟢
Kheiry [7]	2020	🔴	🟡	🔴	🔴	🔴
Thompson [8]	2018	🟡	🟢	🔴	🔴	🔴
Lee [9]	2016	🟡	🟢	🔴	🔴	🔴
Temple [10, 11]	2015, 2016	🟡	🟡	🟢	🟢	🔴
Bender [12]	2013	🟢	🟡	🔴	🔴	🟡
Lee [13]	2013	🟡	🟡	🔴	🔴	🔴
Pepler [14]	2012	🟡	🔴	🔴	🔴	🟡

Hintz [15]	2010	🟡	🟡	🟡	🔴	🔴

Feasibility- Use of real-time data that can be automatically extracted from the electronic health record for decisions at the point of care without additional user input AND without exclusions or data that could only be known in retrospect.

Broadly applicable- Useful across a broad range of the neonatal intensive care unit patient population.

Dynamic prediction- Risk assessments that are updated based on a patient's current condition across the admission, rather than limited to a specified time or feature set.

Actionable- Predicts discharge within a clinically useful window (2 to 10 days).

Rigorously validated- Validated with a population that is separate from development.

🟢 Expectation fully met

🟡 Expectation partially met

🔴 Expectation not met

[*]Elements of *implementable models* as described in Reese TJ, Domenico HJ, Hernandez A, Byrne DW, Moore RP, Williams JB, et al. Implementable prediction of pressure injuries in hospitalized adults: model development and validation. JMIR Med Inform 2024; 12: e51842.

Sources listed in first column

1. Arkin N, Zhao T, Yang Y, Wang L. Development and validation of a novel risk classification tool for predicting long length of stay in NICU blood transfusion infants. Sci Rep 2024; 14: 6877.
2. Erdogan Yildirim A, Canayaz M. Machine learning-based prediction of length of stay (LoS) in the neonatal intensive care unit using ensemble methods. Neural Comput Appl 2024; 36: 14433–14448.
3. Frostig T, Benjamini Y, Kehat O, Weiss-Meilik A, Mandel D, Peleg B, et al. Developing a length of stay prediction model for newborns, achieving better accuracy with greater usability. Int J Med Inform 2023; 180: 105267.
4. Kermani F, Zarkesh MR, Vaziri M, Sheikhtaheri A. A case-based reasoning system for neonatal survival and LOS prediction in neonatal intensive care units: a development and validation study. Sci Rep 2023; 13: 8421.
5. Wang K, Hussain W, Birge JR, Schreiber MD, Adelman D. A high-fidelity model to predict length-of-stay in the neonatal intensive care unit. INFORMS J Comput 2022; 34: 183–195.
6. Singh H, Cho SJ, Gupta S, Kaur R, Sunidhi S, Saluja S, et al. Designing a bed-side system for predicting length of stay in a neonatal intensive care unit. Sci Rep 2021; 11: 3342.
7. Kheiry F, Kargarian-Marvasti S, Afrashteh S, Mohammadbeigi A, Daneshi N, Naderi S, et al. Evaluation of goodness of fit of semiparametric and parametric models in analysis of factors associated with length of stay in neonatal intensive care unit. Clin Exp Pediatr 2020; 63: 361–367.
8. Thompson B, Elish KO, Steele R. Machine learning-based prediction of prolonged length of stay in newborns. 2018 17th IEEE International Conference on Machine Learning and Applications (ICMLA): IEEE; 2018. pp. 1454–1459.
9. Lee HC, Bennett MV, Schulman J, Gould JB, Profit J. Estimating length of stay by patient type in the neonatal intensive care unit. Am J Perinatol 2016: 751–757.
10. Temple MW, Lehmann CU, Fabbri D. Predicting discharge dates from the NICU using progress note data. Pediatrics 2015; 136: e395–e405.

11. Temple MW, Lehmann CU, Fabbri D. Natural language processing for cohort discovery in a discharge prediction model for the neonatal ICU. Appl Clin Inform 2016; 07: 101–115.
12. Bender G, Koestler D, Ombao H, McCourt M, Alskinis B, Rubin LP, et al. Neonatal intensive care unit: predictive models for length of stay. J Perinatol 2013; 33: 147–153.
13. Lee HC, Bennett MV, Schulman J, Gould JB. Accounting for variation in length of NICU stay for extremely low birth weight infants. J Perinatol 2013; 33: 872–876.
14. Pepler F, Uys DW, Nel DG. Predicting mortality and length-of-stay for neonatal admissions to private hospital neonatal intensive care units: a Southern African retrospective study. Afr Health Sci 2012; 12: 166–173.
15. Hintz SR, Bann CM, Ambalavanan N, Cotten CM, Das A, Higgins RD, et al. Predicting time to hospital discharge for extremely preterm infants. Pediatrics 2010; 125: e146–e154.
